# Learning to care for the spirit of dying patients: the impact of spiritual care training in a hospice-setting

**DOI:** 10.1186/s12904-021-00804-4

**Published:** 2021-07-17

**Authors:** Wafie Hussein Chahrour, Niels Christian Hvidt, Elisabeth Assing Hvidt, Dorte Toudal Viftrup

**Affiliations:** Research Unit of General Practice, Department of Public Health, University of Southern Denmark, Odense J. B. Winsløwsvej 9A, 5000 Odense C, Denmark

**Keywords:** Spiritual care, Hospice care, Palliative care, Continuing education, Action research

## Abstract

**Background:**

Patients approaching the end of their life do not experience their existential and spiritual needs being sufficiently met by the healthcare professionals responsible for their care. Research suggest that this is partly due to a lack of insight about spiritual care among healthcare professionals. By developing, implementing, and evaluating a research-based educational course on spiritual care targeting hospice staff, we aimed to explore the perceived barriers for providing spiritual care within a hospice setting and to evaluate the post-course impact among staff members.

**Methods:**

Course development and evaluation was based on primary exploratory action research and followed the UK Medical Research Council’s framework for complex intervention research. The course was implemented at two Danish hospices and comprised thematic days that included lectures, reflective exercises and improvised participatory theatre. We investigated the course impact using a questionnaire and focus group interviews. The questionnaire data were summarized in bar charts and analysis of the transcribed interviews was performed based on Interpretative Phenomenological Analysis.

**Results:**

85 staff members participated in the course. Of these, 57 answered the evaluative questionnaire and 15 participated in 5 focus group interviews.

The course elements that the participants reported to be the most relevant were improvised theatre unfolding existential themes and reflexive group activities. 98% of participants found the course relevant, answering either “relevant” or “very relevant”. 73,1% of participants answered “to a considerable extent” or “to a great extent” when asked to what extent they assessed the content of the course to influence their work in hospice.

The focus group data resulted in 3 overall themes regarding perceived barriers for providing spiritual care: 1. Diverse approaches is beneficial for spiritual care, but the lack of a shared and adequate spiritual language is a communicative barrier, 2. Existential conversation is complicated by patients’ overlapping physical and existential needs, as well as miscommunication, and 3. Providing spiritual care requires spiritual self-reflection, self-awareness, introspection, and vulnerability.

**Conclusions:**

This study provides insights into the barriers facing spiritual care in a hospice setting. Furthermore, the course evaluations demonstrate the valuable impact of spiritual care training for health care professionals. Further course work development is warranted to enhance the “science” of spiritual care for the dying.

**Supplementary Information:**

The online version contains supplementary material available at 10.1186/s12904-021-00804-4.

## Background

The purpose of palliative care is – according to WHO – to prevent and relieve suffering. This includes spiritual care for patients and their families as a field of attention on the same level as physical, social and psychological pain [1]. The European Association of Palliative Care (EAPC), after a comprehensive consensus process that partly built on an earlier north American similar consensus process [2], define spirituality in the following way: “Spirituality is the dynamic dimension of human life that relates to the way persons (individual and community) experience, express, and/or seek meaning, purpose, and transcendence, and the way they connect to the moment, to self, to others, to nature, to the significant, and/or the sacred.” [3]. Spiritual care is understood as a type of care that recognizes and attends to patients’ spiritual needs [4]. Learning to care spiritually for such needs not only involves knowledge of empirical biomedical facts regarding the ailments of a dying patient, but is a “science” in which one is present with the patient in a way that the patient truly experiences (spiritual) care in an interpersonal and authentic manner. Such “science” can be taught and practiced through exercise-based courses, but also grows out of personal experience, attitude and character [5].

According to international research, life-threatening illness might lead to an intensification of spiritual and existential needs and reflections among cancer patients. This intensification progresses in line with disease severity, development, and the prospect of imminent death [6, 7]. Furthermore, research shows that spiritual care can help increase quality of life in the last days among patients who are seriously or terminally ill [8, 9]. To secure comprehensive palliative care, it is therefore necessary to optimize the efforts on meeting the existential and spiritual needs of patients with terminal or life-threatening illness.

Studies find that patients at the end of life do not experience that their existential and spiritual needs are being sufficiently met by the health care professionals responsible for their treatment and care [4]. Research suggests that this is caused by barriers among health care professionals that stem from inadequate knowledge and education on spiritual care, lack of self-reflection and understanding of the existential needs of patients as well as professional shyness when facing spiritual and existential themes in the conversations with patients [10–13].

Denmark is considered one of the least religious nations in the world [14]. Efforts within the area of spiritual care seem to be particularly insufficient in Denmark, which is supported by Danish research pointing to the fact that even religious Danes exhibit a high degree of individualized and private religiosity [15, 16]. However, a Danish study investigating the relationship between health and existential, religious, and spiritual practices through questionnaires from 480 Danish hospital patients at Rigshospitalet in Copenhagen, found positive correlations between the severity of disease and religious, existential, and spiritual practice [17]. These results suggest that even patients in low-religious societies might experience a great need for existential conversation and care when they find their health deteriorating or when they reach the end of life.

Studies indicate that spiritual care training improves health care professionals’ understanding of the theoretical and practical basis of spiritual issues and that it raises awareness of the importance of the spiritual dimension of patient care [18]. A Danish study on the development and evaluation of a course program in existential communication targeting general practitioners, resulted in a significant increase in the participants’ assessed self-efficacy in relation to communicating about existential issues with their patients [19]. Based on these results, we decided to develop and evaluate a similar educational course relevant to and customized for a hospice setting, focusing on spiritual care and communication with patients. The aim of this study was to evaluate the hospice staffs’ experience and self-reported impact of the course in relation to diminishing barriers and enhancing spiritual care to patients at hospice.

## Method

**Fig. 1:**
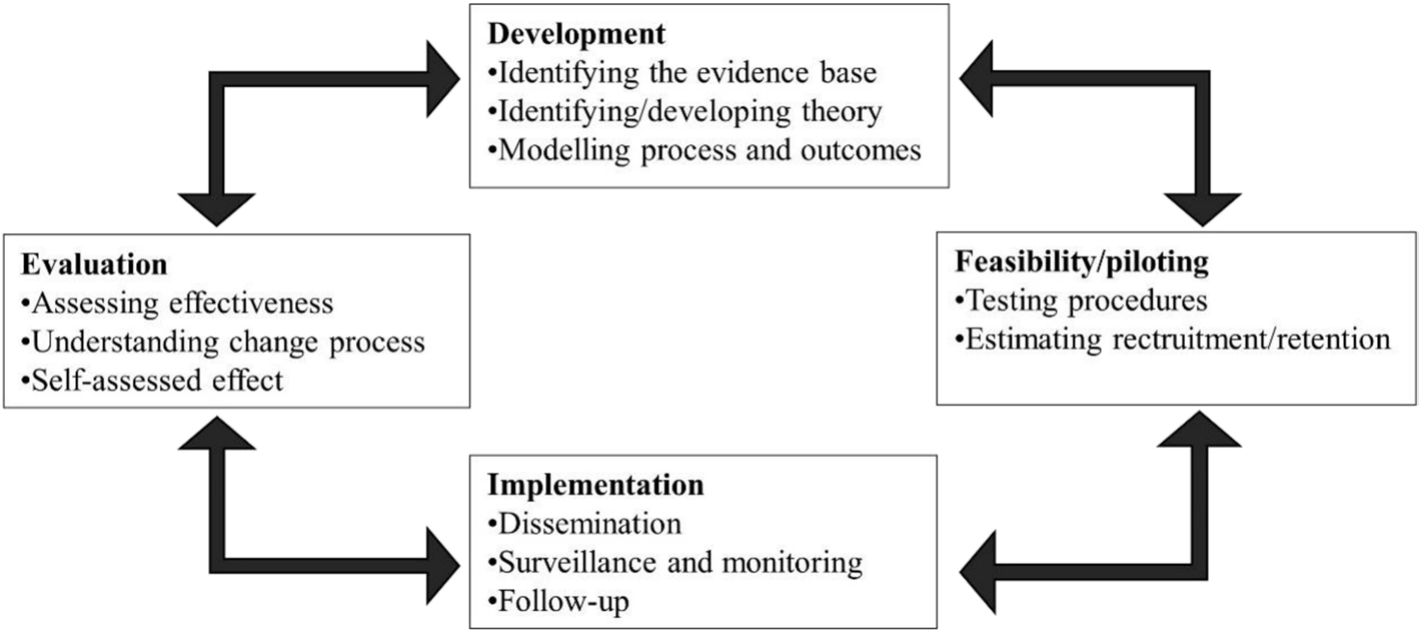
Key elements of the development and evaluation process [20]

The development and evaluation of the educational course followed the outlined framework in the updated guidelines for complex interventions by the UK Medical Research Council (MRC) under the following headings: Development phase, Feasibility and piloting, Evaluation phase and Implementation phase (Fig. 1). The charted phases are a part of a dynamic process, and as such these four stages will usually not follow a linear progress [20].

### Development phase

#### Identifying existing evidence and needs for further explorative research

We found no studies focusing specifically on developing and evaluating an educational course on spiritual care targeting hospice staff when searching the following databases: PubMed, PsychInfo, and Cinahl. In our literature search we opted to only search for studies within a hospice setting to ensure greater comparability to our own study. From a broader point of view, national and international studies have been conducted focusing on educating health care professionals on spiritual care in other health care sectors within the field of palliative care, some of which will be outlined in the following.

In a quantitative study conducted in the Netherlands, an educational course on spiritual care targeting hospital staff was developed and evaluated [21]. The educational course was based on the consensus definition of spirituality by the Taskforce on Spiritual Care of the European Association for Palliative Care (EAPC) and the Dutch guideline on spiritual care. Based on self-reported outcomes from the patients before and after the educational course the study found a significant effect on health care professionals’ attention to patients’ spiritual and existential needs [21]. The same study found a participant self-reported post-course decrease in barriers concerning the provision of spiritual care, although not all participants experienced this difference [22]. Another study with nursing and health care professionals from North West and South West England who underwent spiritual care training evaluated the outcome of the training through individual digital interviews. The qualitative analysis of the transcribed interviews identified two main themes that were defining for the participants’ outcome of the course: *Recognizing spirituality* and *supporting spiritual needs*. These themes encapsulated the impact of spiritual care training on the participants’ clinical roles with subthemes such as *what spirituality means*, *recognition of spiritual distress*, *not having the answers* and *communication skills* [23]. A Danish study developed and evaluated a course programme to enhance existential communication between general practitioners and cancer patients. Similar to our own study, the course was developed based on the UK Medical Research Council’s (MRC) framework for complex interventions, and the evaluation involved both a quantitative and a qualitative evaluation. The results of the study showed a significant increase in mean scores of self-efficacy and the qualitative results suggested a perceived positive significance among participants such as increased existential self-awareness and confidence in the ability to carry out existential communication [19].

Based on the approaches and results of these studies, we conducted primary explorative research in order to develop an educational course relevant for hospice staff in Denmark. The explorative phases of the project were carried out as action research [24], connecting everyday practice and research through participant involvement. A key element within action research is that new knowledge emerges as a result of shared experiences between researchers and participants, and that knowledge is context specific. This requires active researcher involvement in the processes of the field of research with involvement of the participants’ views and experiences. An intervention that is based on action research becomes contextualized to the specific situation as well as the individuals and groups that the intervention is aimed at [25]. The development of the educational course for hospice staff took place in close collaboration with patients and staff at two Danish hospices (Arresødal Hospice and Hospice Sydfyn) and involved observation of clinical work and individual and focus group interviews with patients and staff members. The research methodology was based on existential phenomenology and philosophical hermeneutics [26] which has yielded positive results when used in conjunction with action research in other studies within the field of palliation [27].

### Theoretical framework of the course

The theoretical framework of the course was centred on the following three topics derived from the explorative phases of the project [26]:

1. The vulnerable encounter.
2. Self-reflection concerning personal spiritual needs, thoughts, beliefs, and values.
3. Shared professional language for spiritual care.

*The vulnerable encounter* is about how patients’ needs for spiritual care involves a high degree of relational vulnerability, because addressing spiritual needs and issues often involve a high degree of sensitivity. Both patients and healthcare professionals experience vulnerability in the encounter. For healthcare professionals to enhance spiritual care they must learn to be able to provide spiritual care while being relationally present in the vulnerable encounter. This also relates to the course’s focus on *self-reflection* which other studies has pointed to as a barrier for providing spiritual care [13]. During the explorative phases of the project we found that self-reflection on personal spiritual needs, thoughts, beliefs, and values would positively affect the staff members’ ability to being relationally present in the vulnerable encounter [28]. The theoretical basis for the focus on the vulnerable encounter and self-reflection comes from ethical and relational philosophy [29–32] as well as existential and relational psychology [33–36]. *Shared professional language* involves the importance of shared reflection of spiritual care among the staff members to strengthen spiritual vocabulary in relations to patients as well as facilitating clinically relevant spiritual language for healthcare practitioners. Religion and spirituality in Denmark tends to be privately expressed and less integrated into the vernacular compared to more religious societies [15], which may increase barriers and vulnerability when providing spiritual care. Constructing a shared professional language for spiritual care is expected to decrease barriers.

### Designing the intervention

The course curriculum consisted of three primary elements designed to support the topics of the theoretical framework. Firstly, the participants were introduced to a general understanding of spiritual care in a hospice setting in Denmark. Secondly, they engaged in plenary discussions and exercises that facilitate self-reflection and self-awareness regarding personal spiritual challenges, deliberations, and values. These exercises were based on observations from the explorative phases of the projects and educational elements from “Eksistenslaboratorium” – educational material on existential care aimed at healthcare professionals [37]. Lastly, the curriculum included improvised participatory theatre guided by professional actors. They would act out authentic scenarios from hospice, letting the participants join in or make suggestions about ways to improve or change the interaction. This method has been used to engage healthcare professionals in themes of the vulnerable encounter for decades, and it allows the participants to reflect on their own standpoints in conversation with peers [38]. Improvised participatory theatre also allows the participants to work with existential themes such as meaning, hope, and death [38]. The focus of the immersive improvised theatre was on vulnerability, language use, self-reflection, being present, and caring in the vulnerable spiritual encounter with patients. A detailed overview of the course content is shown in Table 1.

**Table 1:** Overview of the course content

Course curriculum		
	**Curriculum element**	**Content/exercises/activities**
1	Introducing spiritual care in a hospice setting	Introduction to the concept of spiritual care in Denmark and within hospice care Introduction to the concept of Total pain; Physical, Social, psychological, and spiritual pain, and the specific challenges and barriers for spiritual care in a Danish, secular culture
2	Plenary discussions and group exercises	Discussion and reflexive exercises arranged in small groups, or in plenum, and with different instruments, e.g., visual aids or storytelling Topics discussed and reflected on personal values, including spirituality, being present in the pain, the concept of dignity, spiritual self-care, hope, death, and afterlife
3	Improvised participatory theatre	Professional actors playing different fictive scenarios inspired by real events within a hospice setting, involving patients, relatives, and staff members These scenarios involved dilemmas and difficult situations that one can face when providing spiritual care, e.g., angry relatives, patients in great distress, or staff members feeling insufficient in addressing patients’ spiritual needs The staff members could play and rehearse these situations with the actors as well as discussing the difficulties with colleagues hopefully increasing their feeling of self-efficacy

### Deciding on types of evaluation

To evaluate the impact of the educational course, we decided on a combination of quantitative and qualitative evaluation through an anonymous evaluation questionnaire and focus group interviews, respectively. The questionnaire also contained qualitative elements which aimed to elaborate on the quantitative questions as well as allowing the participants to comment on their experiences from the course days with their own words.

The interviews were conducted 2–4 weeks after the course had taken place. They were conducted using a moderator guide with an explorative approach, which was intended to elaborate on relevant aspects of the questionnaire, such as tendencies in the quantitative results, perceived difficulties with implementation of spiritual care in clinical practice, or feedback points regarding the different elements of the course from the qualitative parts of the questionnaire.

The questionnaire [39] was sent out to the participants 2 weeks after completing the course. The quantitative part of the questionnaire covered baseline information such as profession and age. This was followed by questions that allowed the participants to assess, on a Likert scale from 1 to 5 (with 1 being the lowest and 5 being the highest), to what extent they found the different aspects of the course relevant to their everyday work at hospice and to what extent they found themselves better equipped for spiritual conversations with patients. These questions were followed by open-ended questions in which the participants were encouraged to reflect and evaluate on which parts of the course were applicable and relevant, and what needed improvement. This served as the qualitative part of the questionnaire, which aimed to gain a broad and comprehensive understanding of the participants’ experience and self-reported impact of the course.

We decided to perform focus group interviews because we were interested in the participants’ experiences and opinions about the course as a group. This enabled an evaluation of the course that explored the creation of meaning that occurs through a group dynamic [40]. Combining focus group interviews and a questionnaire allowed for method triangulation by using different methods to collect data concerning the same phenomenon [41]. This increased the validity of the study results [40].

### Feasibility and piloting phase

#### Recruitment and participants

All employees (85 individuals) from the two hospices participated in the educational course, both staff with primary patient contact (healthcare professionals) and secondary patient contact (service assistants and administrative staff).

We planned on performing 2–3 focus group interviews at each hospice. We conducted 2 focus group interviews with 4 and 5 participants at one of the hospices. At the other hospice, we conducted 3 interviews with three, two and one participants, respectively, due to busyness at the time of the scheduled interviews. A total of 15 individuals participated in the interviews. The interview with a single participant was performed as an individual interview. All interviews were audio-recorded and transcribed ad verbatim by the first and last author.

### Analysis

The questionnaire data were summarized in bar charts. This was done to better assess the self-reported relevance of the different educational elements on the over-all course as well as the self-reported impact of the educational thematic days. The qualitative elements of the questionnaire consisted mainly of short comments and notes from the course days and did not at such contain comprehensive data. These comments were reviewed with the aim to complement the quantitative data and functioned as informing the focal points for the focus group interviews.

Analysis of the transcribed interviews was performed based on Interpretative Phenomenological Analysis (IPA); a qualitative research methodology committed to the examination of how people make sense of their life experiences [42]. IPA is grounded in phenomenology and hermeneutics. These two philosophical traditions operate under the assumption that human beings create their own narratives, and that the individual’s understanding of different life events is strongly influenced by their interpretations of and emotional response to said events [43]. Through IPA, it is possible to explore nuances as well as shared and opposing experiences concerning the same phenomenon within a small group of participants [43]. This method is particularly relevant to this study as we are interested in a deeper and more nuanced understanding of the perceived barriers that the hospice staff experience in relation to providing spiritual care.

IPA offers a systematic approach to analysing qualitative data in which every case (here: transcribed interview) undergoes a detailed analysis resulting in emergent themes before moving on to analysing the next case. This is followed by a cross-case analysis to find consistency and discrepancies within the emergent themes [44]. Using IPA, we systematically reviewed the interview transcripts and reduced the data material through emerging themes that are pervasive for each interview and across all the interviews. This provided an understanding of the themes in the data material.

The outlined process of this analysis of the data material followed the following steps: Re-reading the data, Initial noting, Developing themes, Searching for connections across themes and Looking for patterns across cases [42]. Methodological rigor was sought upheld through the following analytical processes: The first and last author discussed and compared field observations and preliminary findings continuously throughout the data generation process and in the subsequent systematic coding of the data. Codes and themes were reviewed by all authors in the research group, checking the compliance between them and the meaning in the original transcripts. The definition and naming of the final thematizations were made in the author group on the basis of critical discussions that challenged theoretical assumptions and ideas that might have influenced the way they were generated [45].

## Results

### Evaluation phase

#### Quantitative evaluation

All 85 individuals from the two hospices attended the educational course. Of these, 57 participants answered the evaluative post-course questionnaire, resulting in a response rate of 67%. The mean age, primary or secondary patient contact status and hospice affiliation of the 57 participants is presented in Table 2.

**Table 2:** Age and professional characteristics of participants answering the evaluative post-course questionnaire

Baseline characteristics	
Number of participants answering the evaluative questionnaire, N (%)	57(67)
**Arresødal Hospice**	30 (100)
Mean age, years	52
Primary patient contact, N (%)	23 (79,3)
Secondary patient contact, N (%)	6 (20,7)
**Hospice Sydfyn**	27 (100)
Mean age, years	53
Primary patient contact, N (%)	18 (66,7)
Secondary patient contact, N (%)	9 (33,3)

**Fig. 2:**
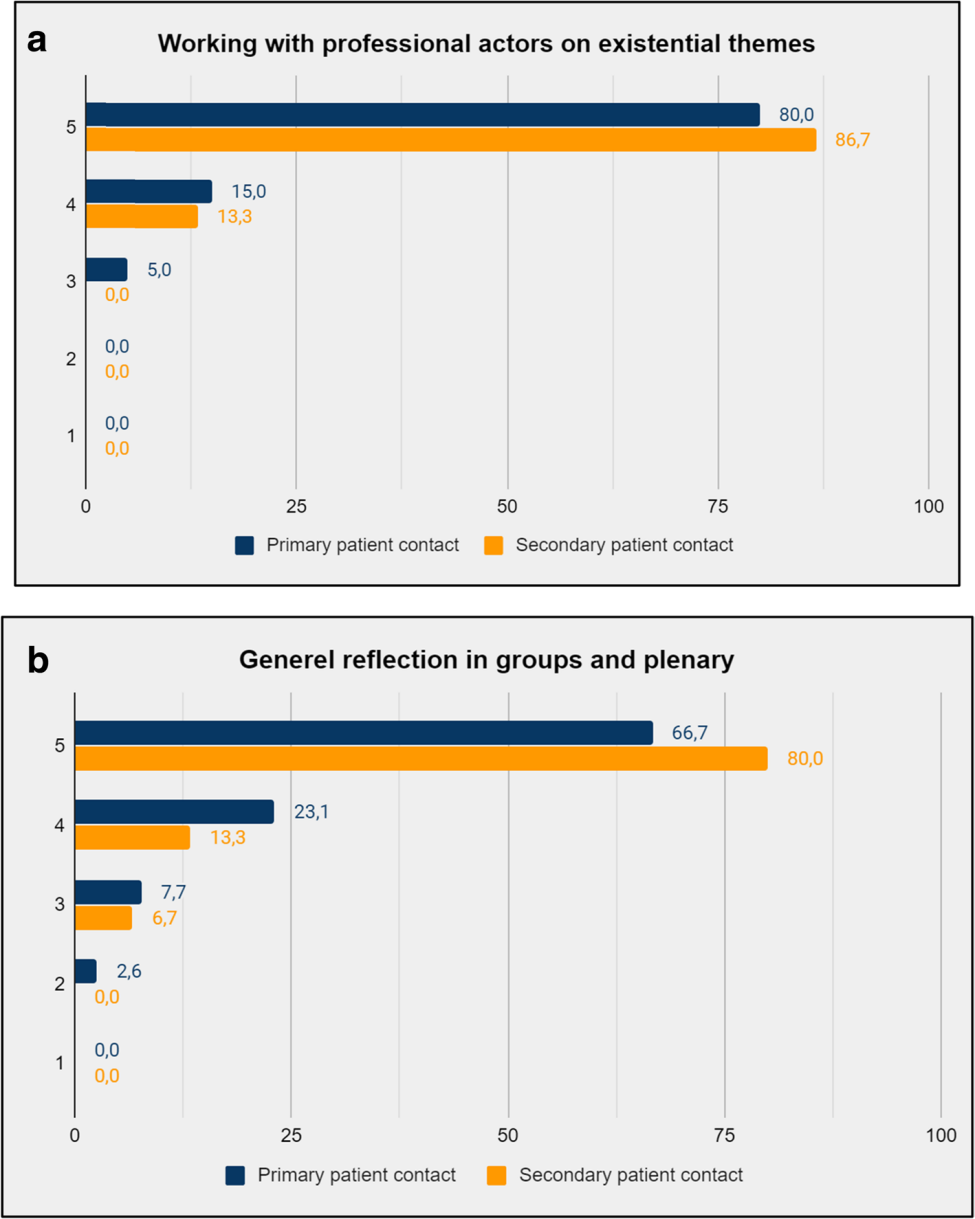
**(a).** Self-reported relevance of working with professional actors on existential themes stated in percent of participants per answer. **(b)**. Self-reported relevance of the reflective group activities stated in percent of participants per answer

**Fig. 3:**
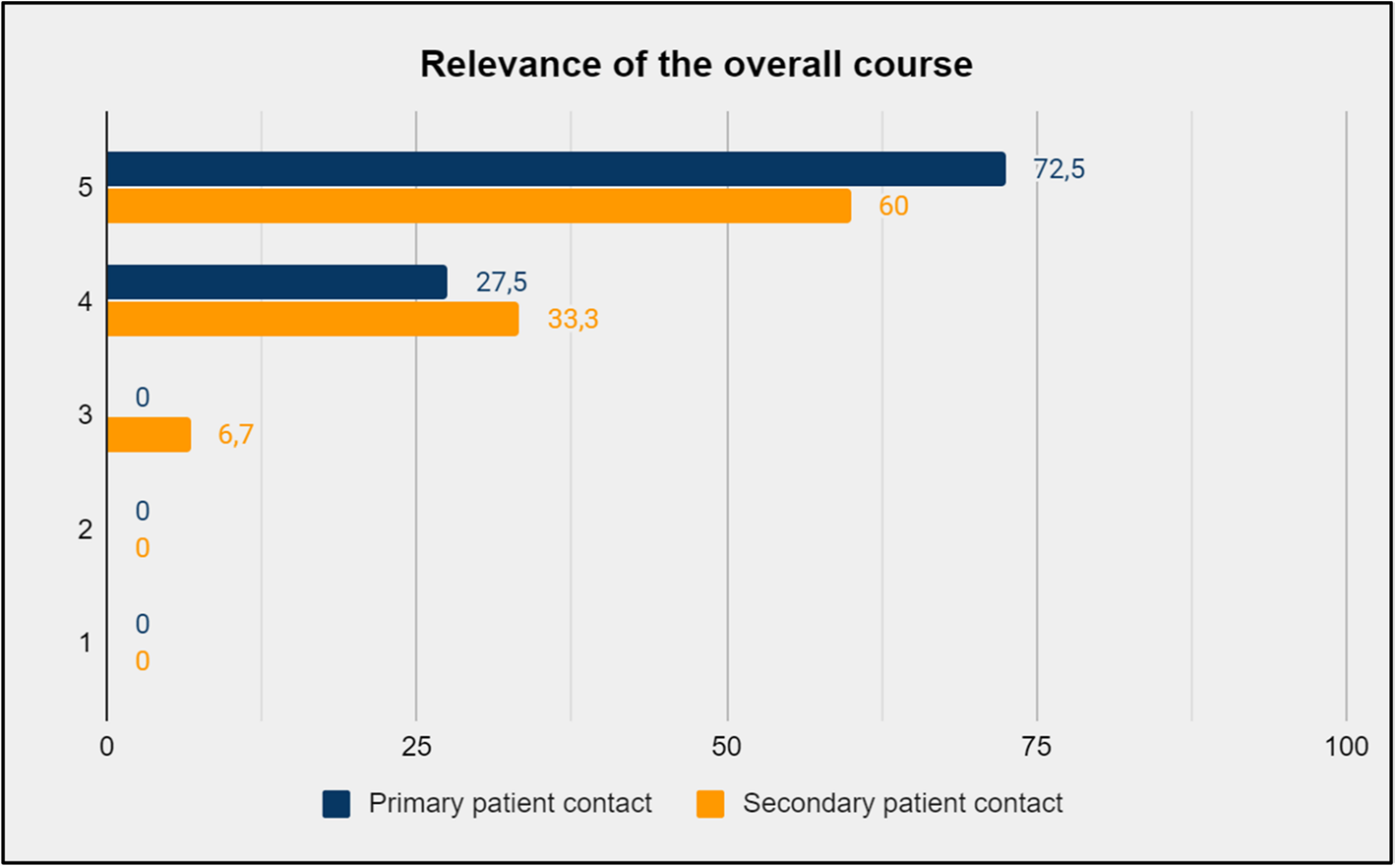
Self-reported relevance of the overall course stated in percent of participants per answer

**Fig. 4:**
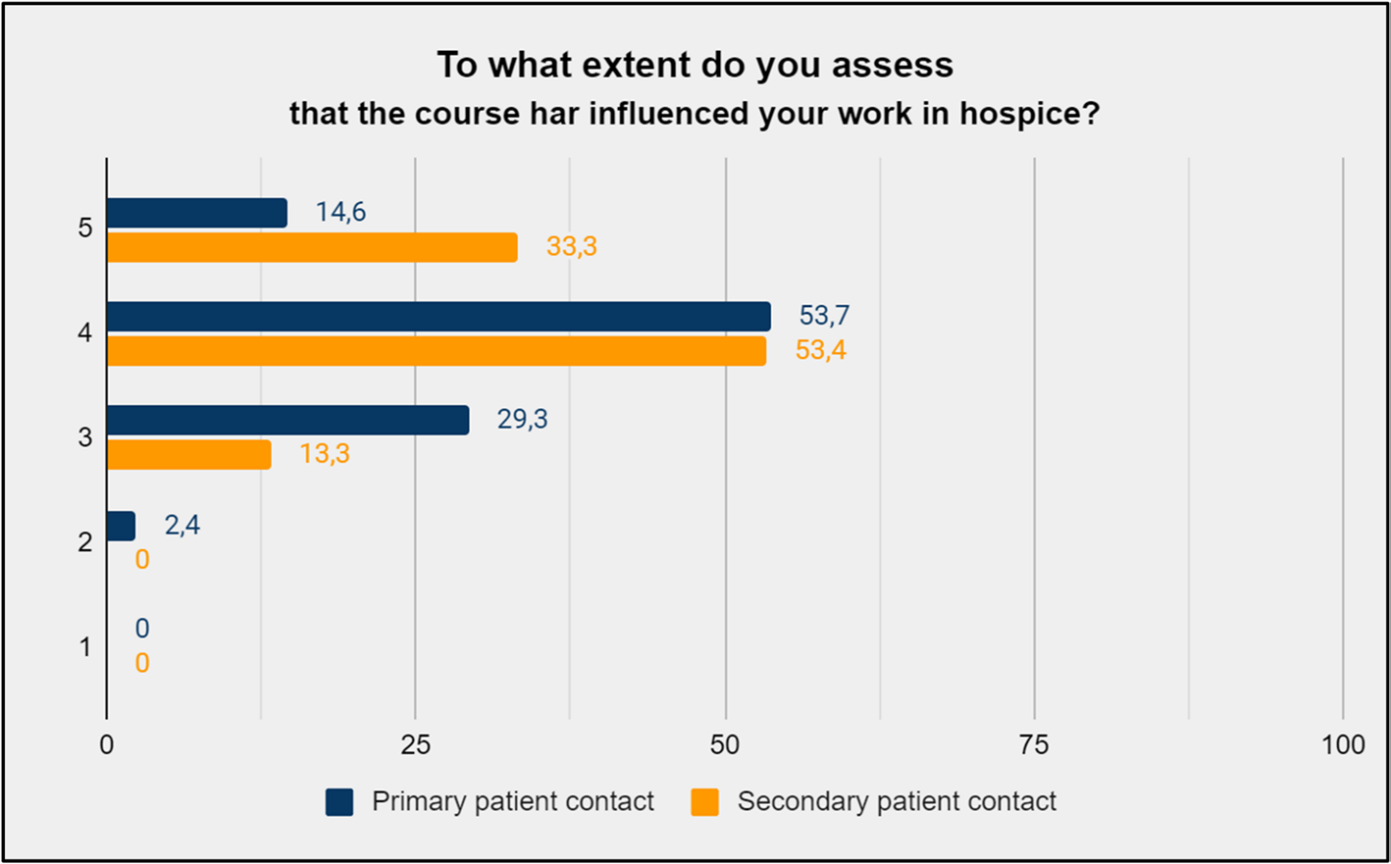
Self-reported influence of the educational course on the everyday work in hospice stated in percent of participants per answer

The educational elements that the participants reported to be the most relevant were improvised theatre unfolding existential themes guided by professional actors and reflective group activities (Fig. 2 (a) and (b)). The overall self-reported relevance of the educational course is presented in Fig. 3. When asked to assess the relevance of the course, 98% answered “relevant” or “very relevant”, corresponding to 4 or 5 on the Likert scale. When asked to what extent they believed the course content would influence their work in hospice, 73,1% of the respondents answered, “to a considerable extent” or “to a great extent”, corresponding to 4 or 5 on the Likert scale. The self-assessed influence of the educational course is shown in Fig. 4.

The participants’ responses in the questionnaire indicate that they found the content and structure of the educational course to be overall rewarding and topically relevant regarding spiritual care. Yet despite this, we see a drop in participants answering 4 or above when it comes to the self-reported effect of the course. This discrepancy between the participants’ experience of the overall relevance of the course and the applicability of the course outcome into a clinical setting may be indicative of an underlying challenge when it comes to implementing the participants’ acquired knowledge and insights in the everyday practice at hospice. When assessing the participants’ experience of the impact of the course, this drop in applicability is a central finding relevant in terms of optimizing future versions of the course programme.

### Qualitative evaluation

The answers to the open-ended questions in the questionnaire were predominately positive with participants for instance commenting: “It was an informative and safe experience, which meant that everybody contributed and said something. We had a valuable exchange of experiences.” and “The different elements of the course complemented each other well, which made the overall course very valuable.” This was in line with the results of the quantitative evaluation data.

When explaining and elaborating on how the course was relevant for their everyday practice in hospice in the questionnaire, some participants found the improvised theatre exercises and cases to be very useful. One participant wrote: “It was education that dealt with exactly the kind of patients we meet every single day. Therefore, it led to some very good reflections and tools to use in our everyday work.” Other participants reported that the course had not directly modified the way they conducted their work at hospice, but it had created increased attention to spiritual care and openness between colleagues in terms of discussing the patients’ spiritual needs. This echoes the discrepancy between the experienced relevance and the clinical applicability of the course found in the quantitative evaluation data. One participant wrote: “I’m not really doing anything differently than I was already doing, but the theme days have offered me better opportunities to talk to the nurses about it [spiritual care] in relation to the patients.”

### Themes

The following three overall themes regarding perceived barriers were developed through the analysis of the transcribed interviews: 1. Diverse approaches is beneficial for spiritual care, but the lack of a shared and adequate spiritual language is a communicative barrier with the subthemes (a) Handling the patients’ existential distress and (b) Strength in diversity, 2. Existential conversation is complicated by patients’ overlapping physical and existential needs, as well as miscommunication and 3. Providing spiritual care requires spiritual self-reflection, self-awareness, introspection and vulnerability.

#### Diverse approaches is beneficial for spiritual care, but the lack of a shared and adequate spiritual language is a communicative barrier

This theme is characterized by the participants’ thoughts and reflections on the practical implementation of spiritual care and communication at hospice. This overall theme comprises two sub-themes: Handling the patients’ existential distress and Strength in diversity.

In the sub-theme *Handling the patient’s existential distress*, the participants reflected on different practical approaches they found important when dealing with patients experiencing existential distress. Some participants emphasized the importance of acknowledging and remembering that it is impossible to fix or find a solution to the existential distress the patients are feeling. One participant expressed the following: “You can help by accommodating, caring, being present, and all of that, but you cannot take it away from someone – all of their feelings and anxiety.” Others pointed to a fear of saying or doing something wrong when dealing with a patient’s existential distress, and how this sometimes could be debilitating in terms of engaging oneself in a vulnerable conversation. A participant said: “It [being afraid of doing something wrong] really is a great hindrance … when you step away from your professionalism and just say here I am, and I’m just going to ask [the patient] – that allows for better communication.” Other participants stated that the most crucial step was starting the conversation with the patient and practicing openness. “You can’t say anything wrong. The worst thing you can do is to say nothing, or to avoid people.” Participants explained how working with the actors had helped them practice different ways to address vulnerability in the conversation and it had further affirmed that there are no finalized answers when engaging in a vulnerable encounter.

This was also echoed within the sub-theme *Strength in diversity*. One participant summarized it as follows: “Is it even possible to define the existential, relational encounter as being right or wrong?”. The diverse approaches to spiritual care and communication practiced by the staff were found to be a strength and “a resource” in the clinical work with patients. Several participants stressed the need for openness towards different personal conducts, when it came to providing spiritual care and communication with the patients. Yet the diversity of opinions and approaches also caused some of the participants to reflect on the need for a shared professional language, and shared ways to express spiritual care – not only with the patients, but especially with each other as colleagues.

#### Existential conversation is complicated by patients’ overlapping physical and existential needs, as well as miscommunication

This theme was developed based on the reservations the participants experienced when addressing existential themes with patients. Course exercises on personal meaning and values were reported to have generated an awareness of the multifaceted connotations and definitions each individual attribute to the notion of spiritual care and communication. One participant noted: “We saw it here – how many different perceptions we had of just a single word.” When viewed in terms of caring spiritually for the existential needs of patients, it became clear that this ambiguity could lead to a distortion of the spiritual conversation with the patient. One participant reflected: “I interpret quite quickly on what others are saying; I know what they mean, because they must mean the same as I do.” Being specific and asking for clarification – “What does this word mean to *you*?” as one participant clarified – was recognised as a helpful and necessary approach to reduce miscommunication while engaging in conversation with patients. Another barrier experienced by some participants was the patients’ difficulty expressing existential distress, and their tendency to focus more on their physical symptoms instead. By extension, it became easier for the staff to direct their questions and care towards the physical wellbeing of the patient instead of the spiritual needs. A participant said “The patient doesn’t always express their existential and spiritual needs. It’s easier to say that your stomach hurts or that you have a headache and it’s easier to question them about that.” Other participants explained that the physical and existential needs often overlap and attending to the patients’ physical health caused “some of the other needs to emerge”. In this way, caring for the patient’s physical state could sometimes become an opening for the caregiver to initiate a conversation on spiritual issues. Some participants recounted situations in which a patient’s physical symptoms had been determined and referred to as ‘existential or terminal distress’ without being addressed further. One participant described it thus: “Sometimes I have asked: and what then? Is it enough to just determine that that the patient’s distress is existential distress?” The participant expressed how they lacked a vocabulary to discuss these gray areas between patients’ physical and spiritual needs sufficiently with their colleagues in order to better navigate and care for the patients.

#### Providing spiritual care requires spiritual self-reflection, self-awareness, introspection and vulnerability

This theme is characterized by the need for spiritual self-reflection and self-awareness among staff to be able to provide sufficient spiritual care for patients, and how that affected them as staff members. After working through the course, several participants recognised spiritual self-reflection and self-awareness as an important step towards bettering the spiritual care offered to the patients at hospice. One participant reflected as follows: “You cannot talk about death if you have not processed the concept of your own death.” They reported that having meaningful conversations with patients in existential distress required a lot of introspection, vulnerability, and honesty on their part. Others found that the personal existential work was important for the process of practising and bettering spiritual care during the course but remained hesitant about implementing this practice into the workplace. One participant said: “My way of regarding the world and other people is also rooted in who I am as a person, but I’m not sure I want to work with my own existential problems at my workplace.”

Course exercises dealing with meaning and values and the inter-collegiate discussions these generated had sparked a wish to introduce this kind of group reflections into the everyday work at hospice for some participants. One participant expressed it as follows: “I find it very rewarding to discuss all these words and terms that we use all the time in our everyday work. We don’t talk about it much from day to day.” Besides being a rewarding way to discuss words and concepts with colleagues, some participants found this method of reflection to be very useful for bringing forth their different opinions and values as they pertain to the patients’ spiritual needs.

## Discussion

### Statement of principal findings

The results from both the quantitative and qualitative analysis of the data indicate that the participants found the content of the educational course useful and relevant for their everyday work. The quantitative results indicated an experience of a drop in applicability of the course outcome into everyday work at hospice. Improvised theatre and group reflections were identified as very successful elements of the course that spurred the discussions and self-reflection. The answers to the open-ended questions and the themes suggest that talking about and working with spiritual care creates an important awareness, both in terms of personal growth and in terms of recognising and managing existential issues and concerns among patients in hospice. The themes developed through the qualitative analysis exhibited complexity and provided insight into the barriers the participants faced in terms of spiritual care provision and how the course has influenced the participants’ approach to spiritual care in continuing phases of their work life.

### Findings in relation to other studies

This project was the first to evaluate the outcome of a research-based educational course on spiritual care targeting hospice staff. Other studies focusing on the outcome of spiritual care training have evaluated on already existing educational courses [23] or courses based on validated guidelines for spiritual care [21].

The quantitative evaluation allowed us to assess the strengths, weaknesses, and the impact of the educational course. The qualitative evaluation provided a nuanced understanding of the difficult nature of spiritual care provision within the context of a hospice setting as experienced by the very people providing it to the patients. Danish projects with the same basic structure as this study [19] support the benefits of conducting both quantitative and qualitative evaluation of a course regarding spiritual care training.

The quantitative results of this study showed that the participants found the educational course to be rewarding, relevant, and informative in terms of providing spiritual care. This was consistent with other studies showing a positive effect on the participants’ attention and approach to patients’ spiritual and existential needs after participating in spiritual care training [19, 21, 22].

An interesting finding in the quantitative data was the drop in applicability of knowledge from the course into an everyday hospice setting, even though the participants found the course rewarding and relevant. Researchers argued that it is difficult to include a spiritual dimension in a clinical setting and that physical care is favored over spiritual care [18]. In our qualitative results we also identified the staffs’ immediate focus on the patients’ physical wellbeing as a barrier for the provision of spiritual care. Research has identified listening and giving the patients time to discuss and explore their existential distress as well as addressing the patients’ spirituality as ways to improve spiritual care in a clinical setting [18].

The analyzed themes in our qualitative evaluation focused on personal self-reflection, the importance of different approaches to spiritual care and the different barriers that the course participants experienced in the face of their patients’ existential and spiritual distress. The findings of other qualitative studies focusing on spiritual care education pointed to similar themes, such as *not having the answers* and *recognition of spiritual distress* [23] as well as *self-reflection and sharing with peers* and *increasing awareness and confidence* [19]. This could indicate that the barriers for spiritual care identified in this study were not isolated experiences at the participating hospices, but rather an expression of prevalent and similar challenges for the provision of spiritual care that healthcare professionals meet throughout the palliative field.

Our qualitative results identified a lack and need for shared professional spiritual language and vocabulary to discuss spiritual care when it came to communicating with both patients and colleagues on spiritual matters. A review focusing on spiritual care training for healthcare professionals also recognized this as a key communicative impediment, which prevented the participants from discussing spiritual care adequately [18]. Creating a shared spiritual language could be complicated when the staff is heavily anchored within the field of biomedicine, which gravitates towards a ‘right or wrong’ way of thinking. This field predominately seeks to ‘fix’ or remove the patient’s pain or distress [46], however, this is often not possible when dealing with spiritual distress. The participants in our study regarded diversity as a resource and an important part of the work with spiritual care in a hospice setting. But including and supporting every individual’s opinions, definitions and approaches to spiritual care might further complicate the creation of shared language and vocabulary.

### Strengths and weaknesses

An important strength of this study was that the development of the educational course was founded in primary, explorative research. However, some limitations apply regarding bias and methodological weaknesses. All staff members who participated in the educational course were encouraged to partake in the subsequent evaluation and to answer the evaluative questionnaire, but not everyone chose to do so. This might give rise to selection bias with an overrepresentation of participants with a mostly positive attitude towards the course and the spiritual content of the course. The interview conducted with a single participant was performed as an individual interview, which meant that important strengths of the focus group method in which the participants would discuss and reflects with their colleagues were lost. The questionnaire focused on the self-reported effect of the course which increased the risk of response bias, as participants could feel inclined to evaluate the course and their abilities in a positive manner. Additionally, the evaluation was only conducted after the educational course, and we therefore have no data from before the course to compare with. Both could result in an overestimation of the self-reported positive impact of the course.

### Implementation phase

Future improvements of this educational course should take further account of how it affects the hospice staff members’ abilities and prerequisites to provide spiritual care for their patients when their starting point is anchored in a biomedical approach and within a biomedical vocabulary. One way to do this during the thematic days could be to further highlight the differences between biomedical and spiritual vocabulary during the improvised theatre scenarios. This could allow the participants to use and create a clinically relevant spiritual language as well as experiencing the different implications of using biomedical and spiritual vocabularies respectively in conversation with patients.

The knowledge derived from this study is meant to lead to an optimization of the educational course and facilitate future implementations and developments. This educational course could be relevant for anyone within the medical field who deals with patients in a life crisis or at the end of life, and as such further implementation may include other hospices, palliative departments, and hospital facilities.

## Conclusion

This study provides insights into the strengths, weaknesses, and the impact of the developed educational course in spiritual care, as well as into the barriers that healthcare professionals face when it comes to providing spiritual care in a hospice setting. Our findings will hopefully contribute to future improvements within the field of spiritual care. Further course work development is warranted to enhance the “science” of spiritual care for the dying.

## Supplementary Information

Additional file 1. English version of questionnaire.

## Data Availability

The datasets used and/or analysed during the current study are available from the corresponding author on reasonable request.
