# Supplementary material for: Learning to care for the spirit of dying patients: the impact of spiritual care training in a hospice-setting
Source: BMC Palliat Care. 2021 Jul 17;20:115. doi: 10.1186/s12904-021-00804-4 (PMC8286591; doi:10.1186/s12904-021-00804-4)
Supplement: Supplementary file 1 — English version of questionnaire. [file 12904_2021_804_MOESM1_ESM.docx]

**Learning to Care for The Spirit of Dying Patients: The Impact of Spiritual Care Training in a Hospice-setting**

Authors: Wafie Hussein Chahrour*, Niels Christian Hvidt**, Elisabeth Assing Hvidt*** Dorte Toudal Viftrup ****

*Corresponding author, BSc. Med., Research Unit of General Practice, Department of Public Health, University of Southern Denmark, Odense J. B. Winsløwsvej 9A, DK-5000 Odense C, Denmark

** ThD., Research Unit of General Practice, Department of Public Health, University of Southern Denmark, Odense J. B. Winsløwsvej 9A, DK-5000 Odense C, Denmark

*** PhD, Mag.art., Research Unit of General Practice, Department of Public Health, University of Southern Denmark, Odense J. B. Winsløwsvej 9A, DK-5000 Odense C, Denmark

***** PhD, M.SC. Psych., Research Unit of General Practice, Department of Public Health, University of Southern Denmark, Odense J. B. Winsløwsvej 9A, DK-5000 Odense C, Denmark*

### **Evaluation of theme days**

The following is the evaluative questionnaire for the theme days. What you write here is fully confidential, all data is anonymised and will be processed according to the instructions of the Data Regulation.

There will be some questions where you have to answer on a scale from 1-5 as well as some where you describe yourself, and I hope you will answer these questions honestly and in depth. The questionnaire takes about 8-10 minutes to complete.

**What theme day did you participate in?**

- Theme day 1
- Theme day 2

**Is your field of work within:**

- Primary patient contact
- Secondary patient contact (e.g. office work, maintenance, management etc)

**What is your age?**

Short answer

**To what extent do you assess that the teaching during the theme days was relevant to work at hospice?**

Please answer below to what extent you found the different educational elements of the theme day relevant for your work at hospice.

1 = Not relevant at all

2 = Not relevant

3 = Neutral

4 = Relevant

5 = Very relevant

**Initial exercise: Life story in 2 minutes**

- 1
- 2
- 3
- 4
- 5

**Internal exercise (in which you reflected in plenary on how you feel in a difficult situation and how you would like to be met in those circumstances)**

- 1
- 2
- 3
- 4
- 5

**Exercise with the significance cards**

- 1
- 2
- 3
- 4
- 5

**Working with actors regarding existential themes (improvised theatre)**

- 1
- 2
- 3
- 4
- 5

**Reflecting over the themes: Being present and accommodation**

- 1
- 2
- 3
- 4
- 5

**The narrative writing-and communication exercise in groups**

- 1
- 2
- 3
- 4
- 5

**General reflection in groups and in plenary**

- 1
- 2
- 3
- 4
- 5

**The overall theme day (overall relevance)**

- 1
- 2
- 3
- 4
- 5

**What did you find most positive about the theme day) Why/how?**

Long answer

**What would have liked to be different? Please elaborate.**

Long answer

**Evaluation regarding practical implementation of the course**

1 = No extent at all

2 = No extent

3 = Neutral

4 = To a considerable extent

5 = To a great extent

**To what extent do you believe the course content has influenced your work in hospice?**

- 1
- 2
- 3
- 4
- 5

**Explain how and in what situation you incorporate what you have learned in your work?**

Long answer

**Evaluation regarding community**

1 = Not at all

2 = Not much

3 = Neutral

4 = A considerable extent

5 = A great extent

**To what extent did you experience that employees were cared for during the theme days?**

- 1
- 2
- 3
- 4
- 5

**To what extent did you experience that there was a good sense of community with you colleagues?**

- 1
- 2
- 3
- 4
- 5

**Do you have anything else to add regarding the theme day?**

- 1
- 2
- 3
- 4
- 5
